# Hemophagocytic Lymphohistiocytosis Complicating Dengue and *Plasmodium vivax* Coinfection

**DOI:** 10.1155/2015/696842

**Published:** 2015-10-04

**Authors:** Muhammad Khurram, Muhammad Faheem, Muhammad Umar, Asif Yasin, Wajeeha Qayyum, Amna Ashraf, Javeria Zahid Khan, Ali Hasnain Yasir, Yusra Ansari, Muhammad Asad, Iram Khan, Shuja Abbas, Irum Rasheed, Natasha Rasool, Hamama Tul Bushra Khar

**Affiliations:** Rawalpindi Medical College, Holy Family Hospital, Rawalpindi 46300, Pakistan

## Abstract

Hemophagocytic lymphohistiocytosis (HLH) is a rare disorder. Dysfunction of cytotoxic T and natural killer (NK) cells causes uncontrolled activity of lymphocytes and histiocytes which leads to HLH. Infections, malignancies, and autoimmune disorders are associated with development of HLH. Dengue and *Plasmodium vivax* are rare causes of HLH. We report the first ever case of a young man who developed fatal HLH that complicated Dengue Hemorrhagic Fever (DHF) and *Plasmodium vivax* infection.

## 1. Introduction

Hemophagocytic lymphohistiocytosis (HLH) is a rare disorder which can be familial or acquired. Acquired HLH complicates a number of viral, bacterial, and parasitic infections. It is also associated with autoimmune disorders and certain malignancies like T cell lymphoma. Familial HLH has autosomal recessive transmission and is seen in infants less than 18 months old. HLH is associated with high mortality [1]. HLH results from dysfunction of cytotoxic T and natural killer (NK) cells that cause uncontrolled lymphocytes and histiocytes activity which is associated with phagocytosis of hematopoietic cells [2].

HLH is characterized by prolonged fever and sepsis-like syndrome. It presents with nonspecific clinical features like fever and hepatosplenomegaly. Laboratory findings include cytopenias, raised serum aminotransferase levels, hypofibrinogenemia, hypertriglyceridemia, hyperferritinemia, and increased lactic dehydrogenase (LDH) levels. Dengue and malaria are important but rare causes of HLH [3, 4]. We report the first ever case of 19-year-old male with HLH diagnosed in settings of both Dengue Hemorrhagic Fever (DHF) and* Plasmodium vivax* (*P*.* vivax*) malaria infections.

## 2. Case Report

A 19-year-old, previously healthy, motor mechanic was admitted with 13-day history of high grade fever, headache, retroorbital pain, and myalgias. One day before admission he became restless and irritable. On examination his pulse was 104/minute, temperature 101°F, respiratory rate 24/minute, and blood pressure 110/70 mmHg (pulse pressure 40 mmHg). He was jaundiced. Abdominal examination revealed tender hepatomegaly with liver span of 18 cm. Chest examination was suggestive of right sided mild to moderate pleural effusion. He was noted to be drowsy, disoriented, and confused. His Glasgow Coma Scale (GCS) was 12/15 (E3, M5, and V4). No focal neurological deficit or signs of meningeal irritation were noted. The rest of the clinical examination was unremarkable.

Ultrasonographic examination at admission showed thick walled gall bladder (wall thickness 13 mm), hepatomegaly (17 cm), splenomegaly (13.6 cm), mild to moderate ascites, and mild to moderate pleural effusion. CT scan brain without contrast showed brain edema. Respiratory alkalosis was noted on arterial blood gas (ABG) analysis. ECG was normal and chest X-ray showed right pleural effusion. Other investigations are shown in Table 1.

*Day 1*. An initial assessment of DHF complicated by dengue fulminant hepatic failure was made with differential diagnosis of encephalitis, cerebral malaria, sepsis, and multiorgan dysfunction. Injectable artesunate, piperacillin/tazobactam, acyclovir, and dexamethasone (0.4 mg/Kg, 12 hourly) were started. Standard DHF management was commenced. Lactulose and N-acetyl cysteine were also administered per nasogastric tube.

*Day 2*. Clinical condition remained the same. Investigation showed positive dengue markers (NS1, IgM antibodies, and IgG antibodies performed by SD Dengue Capture ELISA Kit) and positive smear examination for malarial parasite (gametocytes and schizonts of* P*.* vivax*). Markers for hepatitis A, hepatitis B, hepatitis C, and hepatitis E turned out negative. Ferritin (40000 ng/mL, normal value 12–300 ng/mL) and triglycerides levels (292 mg%, normal value < 140 mg/dL) were elevated. Serum fibrinogen levels were on lower side (Table 1). Diagnosis of HLH in settings of DHF and* P*.* vivax* coinfection was considered. Dexamethasone was replaced with methyl prednisolone (30 mg/Kg/day for 3 days). PCR for dengue was also sent along with Congo fever markers.

*Day 3*. Patient's conscious level, restlessness, and irritability improved. Two episodes of melena and epistaxis occurred. Intravenous proton pump infusion was started and fresh whole blood was transfused.

*Day 4*. Patient became breathless. Chest examination revealed bilateral wheeze and crepitations. Oxygen saturation dropped to 74%. On ABGs, PO_2_ was 48 mmHg. CXR showed bilateral infiltrates. Diagnosis of ARDS was considered and patient was started on ventilatory support.

*Day 5*. Patient's condition remained the same. Mechanical ventilation was continued. ECG showed sinus tachycardia. Dexamethasone was restarted at a dose of 10 mg/m^2^ per day.

*Day 6*. Fever and hypotension developed. Antibiotics were modified to Imipenem and Vancomycin considering the diagnosis of hospital acquired infection. Ultrasound scan revealed right sided moderate to massive pleural effusion. Thoracocentesis was done and straw-colored 1-liter fluid was aspirated which was transudative on laboratory evaluation. Echocardiography showed dilated left ventricular (LV) and globally reduced LV systolic function. Ejection fraction was 25%. Diagnosis of dengue myocarditis was also considered and digoxin was added to treatment regimen.

**Figure 1:**
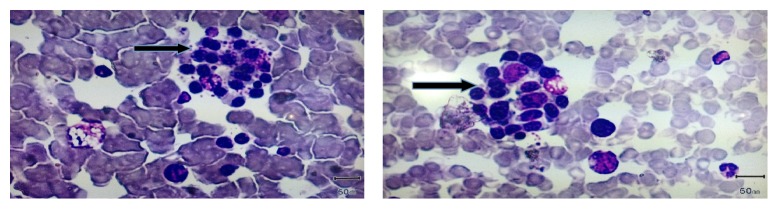
Two bone marrow biopsy slides of patient showing histiocyte surrounded by erythroblasts (arrow).

*Day 7*. Patient's condition did not improve and management continued. Repeat ultrasound scan showed mild bilateral effusion. Bone marrow biopsy was done; it was suggestive of HLH (Figure 1).

*Days 8 and 9*. Patient went into asystole and resuscitation resulted in recovery. Inotropic support was added.

*Day 10*. Patient expired.

The report of dengue PCR and Congo fever markers was received after expiry. Den 3 was isolated (RNA extraction by Qiagen viral RNA mini kit, and amplification by real-time PCR). Congo fever markers were negative.

## 3. Discussion

HLH is an uncommon inflammatory disorder which is characterized by activation of macrophages that cause phagocytosis of blood cells in bone marrow. HLH is diagnosed when 5 out 8 diagnostic criteria are fulfilled [2, 5–7]. Criteria fulfilled in our patients were (1) fever, (2) cytopenia on peripheral film examination, (3) hypertriglyceridemia or hypofibrinogenemia, (4) hyperferritinemia, and (5) enlargement of spleen. Bone marrow picture in our patient was suggestive but not diagnostic of HLH. It should however be noted that, in about 30% of patients with HLH, first bone marrow examination may not be diagnostic and repeat biopsy may have to be performed [8]. We did not get it; additionally evaluation of natural killer (NK) cell activity and soluble interleukin (IL) level was not possible in our circumstances.

Dengue is an uncommon cause of HLH [9–12]. In dengue infection, virus infected T cells produce cytokines like TNF-*α* and IFN gamma which possibly contribute to development of HLH syndrome [13]. Most of the dengue related HLH cases described in literature are associated with DHF [4]. Our patient had DHF: (1) his illness started during dengue epidemic period in a dengue epidemic hit area, (2) clinical features were suggestive of dengue infection, that is, fever, headache, retroorbital pain, and myalgias, (3) thrombocytopenia was noted, (4) serological tests for secondary dengue infection and PCR were positive, and (5) ultrasonography showed evidence of plasma leakage on day 1. In our patient DEN 3 was isolated. DEN 3 has been noted to be associated with HLH in USA [14]. HLH due to DEN 1 and DEN 4 was noted in Puerto Rico [15].

*P. vivax* malaria is another unusual cause of HLH syndrome [5, 11]. Malarial infection causes increased production of interferon gamma, tumor necrosis factor-alpha, interleukin-1, and interleukin-6 that may lead to HLH [13]. Fever, enlargement of spleen, and thrombocytopenia in our patient indicate, while* P. vivax* detection confirms diagnosis of malaria.

Treatment of the cause, supportive therapy, and suppressing immune response are main stay of HLH management [16]. Specific treatment of HLH is based on HLH protocol that includes usage of dexamethasone, etoposide, and intrathecal methotrexate [16]. It is further divisible in induction, salvage, and continuation therapies. Antithymocyte globulin or alemtuzumab, anti-interferon-*γ* monoclonal antibodies, and hematopoietic stem cell transplantation are additional available modalities for HLH treatment [16].

Complexity of clinical situation, difficulty in differentiation from sepsis, and multiorgan dysfunction lead to delayed diagnosis in HLH [16]. Untreated HLH is associated with poor outcome. In a study focusing on 162 adult HLH patients, 58% survival was noted [8]. In a seminar about adult hemophagocytic syndrome 41% mortality was described in 1109 adult patients [5]. Outcome in* P*.* vivax* associated HLH is generally good if appropriate antimalarials are used [17–19]. 0–100% mortality has been reported in dengue related HLH [9, 10, 15, 20]. Dengue related HLH in which the patient recovered has been documented in Pakistan as well [21].

Our patient had dual infection with dengue virus and* P*.* vivax*. Malaria and dengue coinfection can occur in countries where these are endemic; however HLH complicating the two infections has never been reported according to our knowledge. Both these diseases come in the differential diagnosis of acute febrile illness. The question is what predominantly caused HLH in our patient.* P. vivax* was not detected after artesunate administration on smear and bone marrow examinations of our patient making it less plausible etiological factor for HLH development.

Persistence of fever > 8 days in dengue alerts towards diagnosis of HLH, as duration of dengue febrile phase is generally 3–7 days [22, 23]. Additionally dengue virus remains detectable for 2–12 days after the onset of illness [24]. In our patients patient dengue virus was isolated on 14th day of onset of illness which is unusual and has not been focused previously. HLH thus possibly complicated dengue infection. Genetic factors and* P. vivax* infection may have additional variable contribution.

Our patient had cardiac involvement. Troponin T was negative twice however. It is known that Troponin autoantibodies can result in false negative Troponin T results [25]. Was it uncontrolled immune activity which lead to it in our patient? This remains to be evaluated in further studies.

Our patient received focused DHF management, antimalarials for* P. vivax*, corticosteroids for HLH, cover for infection, and supportive therapy. He however did not receive etoposide, methotrexate, and immunoglobulins. HLH related neurological, respiratory, hepatic, and cardiac involvement lead to multiorgan dysfunction which caused death in our patient. Infection possibly contributed to it as well, which can complicate the scenario because of HLH pathophysiology and immunosuppressive medications like steroids used in treatment. HLH protocol was not employed in our patient that may have altered the outcome. Remembering that HLH can occur in similar settings can help in early diagnosis and institution of HLH protocol.

## Figures and Tables

**Table 1:** Hematological and biochemical parameters.

	Day 1	Day 2	Day 3	Day 4	Day 5	Day 6	Day 7	Day 8
Hemoglobin (g%)	13.4	7.8	9.3	9.4	10.3	12.5	14.0	12.9
Hematocrit (%)	38.9	22.5	26.6	26	29.4	37.3	40.0	39
WBC (×10³/cu mm)	17.2	5.3	6.3	3.1	7.0	8.4	6.2	5.4
Neutrophils (%)	25.9	45.5	53.7	70.3	84.1		79	75
Lymphocytes (%)	70.3	47.5	40	25.3	8.7		17	20
Platelets (×10³/cu mm)	80	62	74	69	78	138	258	229
PT^*∗*^ (seconds prolonged)		7	6	0			8	2
aPTT^*∗∗*^ (seconds prolonged)		7	37	12			2	2
Fibrinogen (150–350 mg/dL)		160	135					225
ALT^*∗∗∗*^ (<43 IU/L)	1283	684	865	546	486	343	284	291
AST^*∗∗∗∗*^ (<43 IU/L)	37						64	
ALP^*∗∗∗∗∗*^ (<147 IU/L)	1974							
Bilirubin (<1.0 mg/dL)	8.3	5	5.1			3.8		
Albumin (3.5–5 g/dL)	4.4	3.2	3.3			4.6	4.1	
Urea (10–50 mg/dL)	75	52	45	90	39	36	42	
Creatinine (<1.2 mg/dL)	1	0.9	0.4	1.0	0.8	0.8	0.9	
Na^+^ (135–145 meq/L)	129	137	135	133		135	144	
K^+^ (3–5 meq/L)	5.6	4.3	4.0	3.2			4.5	
Calcium (8–10 mg/dL)	9.6	7.9	8.6	7.0	7.2	6.8	8.1	9.1
Amylase (30–110 U/L)	175	260	230					
Serum lipase (<50 IU/L)						74.6		
Creatinine kinase (<190 IU/L)		1668	1355					
Serum LDH (225–450 IU/L)	123	535					60	
Troponin T (by ICT)	Negative					Negative		

^*∗*^PT, prothrombin time; ^*∗∗*^aPTT, activated partial thromboplastin time; ^*∗∗∗*^ALT, alanine transferase; ^*∗∗∗∗*^AST, aspartate transferase; ^*∗∗∗∗∗*^ALP, alkaline phosphatase.
